# Unilateral laminectomy for bilateral decompression improves low back pain while standing equally on both sides in patients with lumbar canal stenosis: analysis using a detailed visual analogue scale

**DOI:** 10.1186/s12891-019-2475-6

**Published:** 2019-03-04

**Authors:** Hiroshi Takahashi, Yasuchika Aoki, Junya Saito, Arata Nakajima, Masato Sonobe, Yorikazu Akatsu, Masahiro Inoue, Shinji Taniguchi, Manabu Yamada, Keita Koyama, Keiichiro Yamamoto, Yasuhiro Shiga, Kazuhide Inage, Sumihisa Orita, Satoshi Maki, Takeo Furuya, Masao Koda, Masashi Yamazaki, Seiji Ohtori, Koichi Nakagawa

**Affiliations:** 1 Department of Orthopaedic Surgery, Toho University Sakura Medical Center, 564-1, Shimoshizu, Sakura, Chiba 285-8741 Japan; 2 Department of Orthopaedic Surgery, Eastern Chiba Medical Center, Togane, Japan; 3 Department of Orthopaedic Surgery, Chiba University Graduate School of Medicine, Chiba, Japan; 4 Department of Orthopaedic Surgery, Faculty of Medicine, University of Tsukuba, Tsukuba, Japan

**Keywords:** Lumbar spinal stenosis, Unilateral laminectomy for bilateral decompression, Visual analogue scale

## Abstract

**Background:**

Unilateral laminectomy for bilateral decompression (ULBD) for lumbar spinal stenosis (LSS) is a less invasive technique compared to conventional laminectomy. Recently, several authors have reported favorable results of low back pain (LBP) in patients of LSS treated with ULBD. However, the detailed changes and localization of LBP before and after ULBD for LSS remain unclear. Furthermore, unsymmetrical invasion to para-spinal muscle and facet joint may result in the residual unsymmetrical symptoms. To clarify these points, we conducted an observational study and used detailed visual analog scale (VAS) scores to evaluate the characteristics and bilateral changes of LBP and lower extremity symptoms.

**Methods:**

We included 50 patients with LSS treated with ULBD. A detailed visual analogue scale (VAS; 100 mm) score of LBP in three different postural positions: motion, standing, and sitting, and bilateral VAS score (approached side versus opposite side) of LBP, lower extremity pain (LEP), and lower extremity numbness (LEN) were measured. Oswestry Disability Index (ODI) was used to quantify the clinical improvement.

**Results:**

Detailed LBP VAS score before surgery was 51.5 ± 32.5 in motion, 63.0 ± 30.1 while standing, and 37.8 ± 31.8 while sitting; and showed LBP while standing was significantly greater than LBP while sitting (*p* < 0.01). After surgery, LBP while standing was significantly improved relative to that while sitting (*p* < 0.05), and levels of LBP in the three postures became almost the same with ODI improvement. Bilateral VAS scores showed significant improvement equally on both sides (*p* < 0.01).

**Conclusions:**

ULBD improves LBP while standing equally on both sides in patients with LCS. The improvement of LBP by the ULBD surgery suggests radicular LBP improved because of decompression surgery. Furthermore, the symmetric improvement of LBP by the ULBD surgery suggests unsymmetrical invasion of the paraspinal muscles and facet joints is unrelated to residual LBP.

## Background

Lumbar spinal stenosis (LSS) is one of the most common disorders affecting the elderly. Spinal surgery is well known to be required as a treatment for patients with LSS who fail to respond to conservative treatment. Conventional laminectomy is the most widespread surgery for LSS and removes the posterior structures including the lamina, spinal processes, spinous ligament, and medial facet joints. However, conventional laminectomy can result in postoperative back pain due to the instability of vertebra after destruction of the posterior structures [1, 2]. Therefore, less invasive surgery such as unilateral laminectomy for bilateral decompression (ULBD) as first introduced by Young et al. has been studied [3]. Favorable results for low back pain (LBP) and lower extremity pain (LEP) and numbness (LEN) in patients with LSS treated with ULBD have been demonstrated [4–8]. In most of those studies, a conventional visual analog scale (VAS) score and Oswestry disability index (ODI) were used to evaluate LBP. However, there are few reports of the detailed changes and localization of LBP before and after ULBD for LSS. Furthermore, unsymmetrical invasion of paraspinal muscles and laminae may result in unsymmetrical residual LBP. To clarify these points, we conducted an observational study and used detailed VAS scores to evaluate the characteristics and bilateral changes of LBP, LEP, and LEN.

## Methods

### Patient population

The present study was approved by our hospital’s human ethics committee. Informed consent was obtained from all patients. We enrolled 74 patients treated with ULBD for LSS from April 2010 to April 2016. LSS was diagnosed by two orthopedic spine surgeons from neurological findings, and persistent and unremitting LBP for more than 3 months, X-ray images, and magnetic resonance imaging (MRI). All the patients were treated with sufficient conservative treatment and those who were not improved by conservative treatment and wished to undergo the surgical treatment were included in this study. Patients who did not wish to undergo fusion surgery or who underwent ULBD complicated with degenerative spondylolisthesis (DLS) having instability of vertebra were excluded. The definition of instability was as follows: sagittal translation of 8% or more on flexion–extension lateral X-ray images, anterior wedging of 5° or more on flexion X-ray images, or a disc range of motion of 10° or more, as consistent with past reports [9]. Double lesion cases (complicated with cervical spondylotic myelopathy and LSS) were also excluded. As a result, 4 cases of DLS and 4 cases of double lesion were excluded. In addition, 10 cases were excluded because of lack of data and 6 cases because of loss to follow-up. Ultimately, 50 cases were included in the present study.

### ULBD

All the operative procedures were performed under a surgical microscope as described in detail elsewhere [3, 4, 7]. In brief, under general anesthesia, the patient was placed in a prone position. After the confirmation of the decompression level using an X-ray image, the level of skin incision was determined. Only the interlaminar spaces of the approached side were exposed with the dissection of paraspinal muscles from the midline. Hemilaminectomy of the approached side was performed by removing the inferior aspect of the cranial hemilamina, superior aspect of the caudal hemilamina, and a portion of the medial facet using a high-speed burr and an osteotome. The ligamentum flavum on the approached side was removed and the dural sac and the nerve root on the approached side were decompressed. After the hemiflavectomy on the approached side, the operating table was tilted down and the surgical microscope was angled for decompression of the opposite side. After removing the deep cortical surface, the cranial hemilamina of the opposite side was removed using a high-speed burr and an osteotome. Finally, the ligamentum flavum of the opposite side was removed and the dural sac and nerve root of the opposite side were decompressed. The spinous process and supraspinous and interspinous ligaments were preserved. Generally, the more symptomatic side was chosen as the approached side, and if the symptoms were similar in each side, a left-side approach was chosen. In the recent 8 cases of single level decompression since 2015, the surgeries were performed using a tubular retractor (METRx MD system, Medtronic, US) for even less invasive damage to paraspinal muscles on the approached side.

### Evaluation of low back pain, lower extremity pain, and numbness

**Fig. 1:**
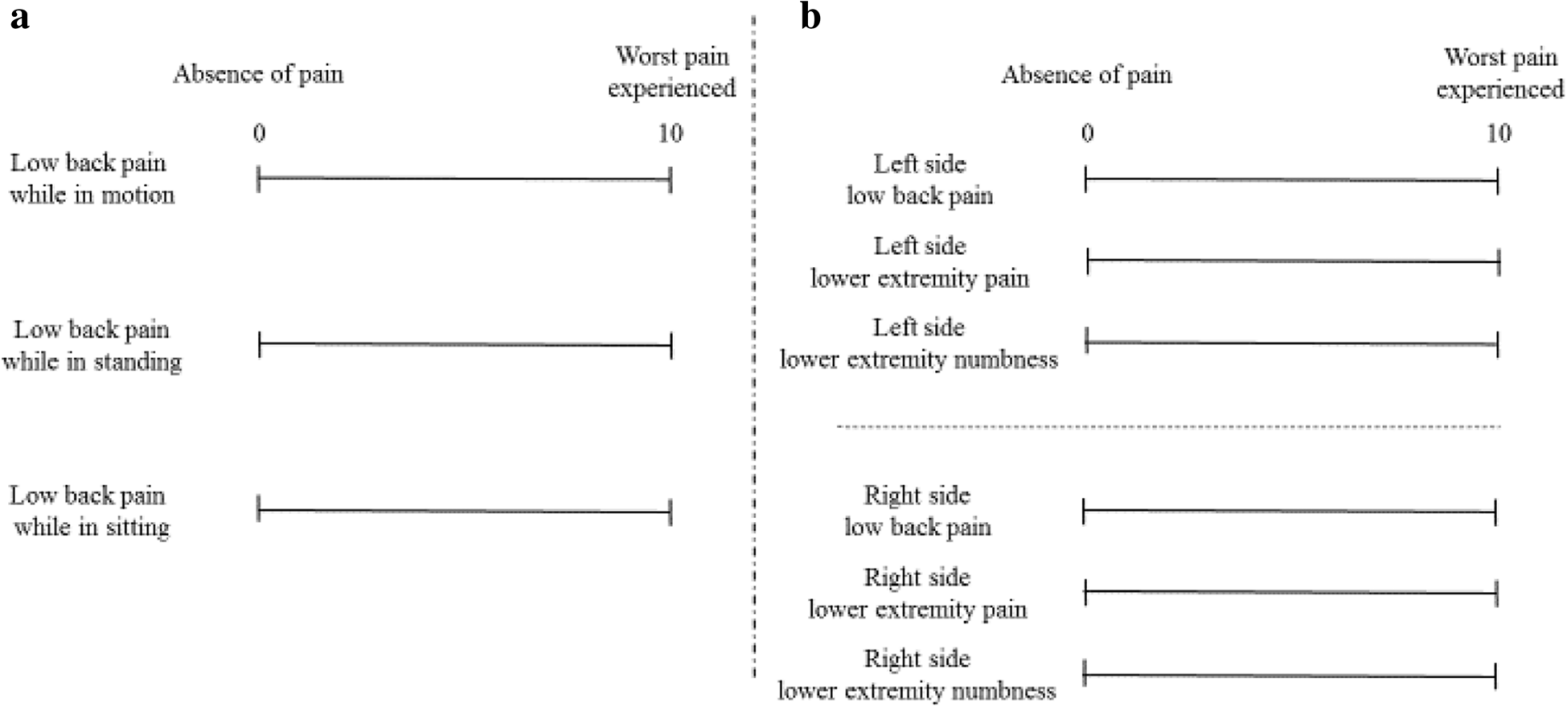
VAS scores. **a** Detailed LBP VAS (0–100 mm) scores introduced by Aoki et al. The LBP was scored independently in three different postural situations: motion, standing, and sitting. **b** LBP, LEP, and LEN VAS (0–100 mm) scores bilaterally on the approached and opposite sides

The definition of LBP in this study did not include buttock pain. First, we measured detailed VAS (100 mm) score as developed by Aoki et al. for LBP in three postural situations: motion, standing, and sitting (Fig. 1a) [10]. Second, we evaluated localization (left side versus right side) of LBP, LEP, and LEN measuring the VAS score bilaterally on the approached and opposite sides (Fig. 1b). Bilateral VAS scores were evaluated. ODI was measured to check the activity of daily living and clinical improvement. All VAS scores and ODI values were measured before surgery, and at 6 months, 1 year, and 2 years’ follow-up.

### Evaluation of imaging findings

We investigated X-ray lateral image functional views before surgery and at the 2-year follow-up. Changes of local range of motion (ROM) and translation in the decompression segment were measured and correlations with the detailed LBP VAS score were evaluated. In the multilevel decompression cases, the maximum values of the decompression segment were investigated.

### Statistical analyses

Results are presented as mean ± standard deviation. One factor ANOVA with a post hoc Tukey–Kramer test was used to determine differences between the three postural VAS scores. A paired *t* test was used to determine the improvement of each VAS score after surgery. A repeated measures ANOVA with a post hoc Turkey–Kramer test was used to determine changes in VAS scores bilaterally and ODI. *P* < 0.05 was considered significant in the tests of statistical inference. All statistical analyses were performed using SPSS (ver. 21) software (IBM Corporation, Armonk, NY, USA).

## Results

### Patient characteristics

**Fig. 2:**
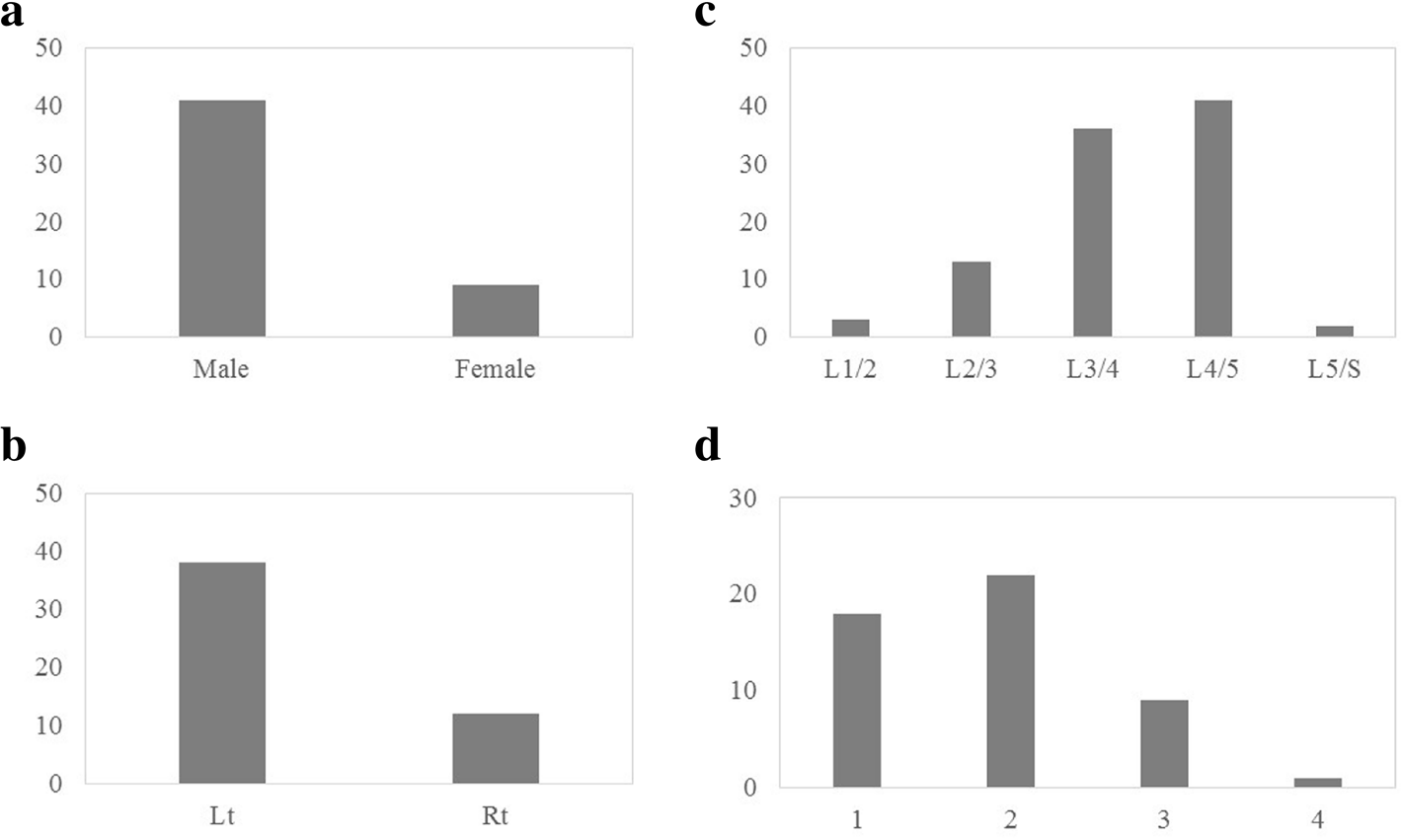
Patient characteristics in 50 cases. **a** Sex. **b** Approached side, Lt: left side approach. Rt: right side approach. **c** Decompression level (L1–2 to L5-. **d** Levels decompressed

The characteristics of the 50 patients are shown in Fig. 2. Their mean age was 70.1 ± 7.97 years. The proportion of women was higher than that of men (Fig. 2a). The left side approach was more frequent because the surgeons in this series were right handed and the left side approach was chosen if the symptoms were similar on both sides (Fig. 2b). ULBD was used on 95 levels. The proportions of L3–4 and L4–5 were more frequent in the decompression level and the proportions of L5-S were less frequent because cases of lumbar disc herniation were excluded from this series (Fig. 2c). Single level and two levels surgery were frequently used for decompression (Fig. 2d). There were no cases of surgical site infection in this series, nor were there other critical complications such as thromboembolic events or severe neurological deficits.

### Evaluation of VAS and ODI

**Fig. 3:**
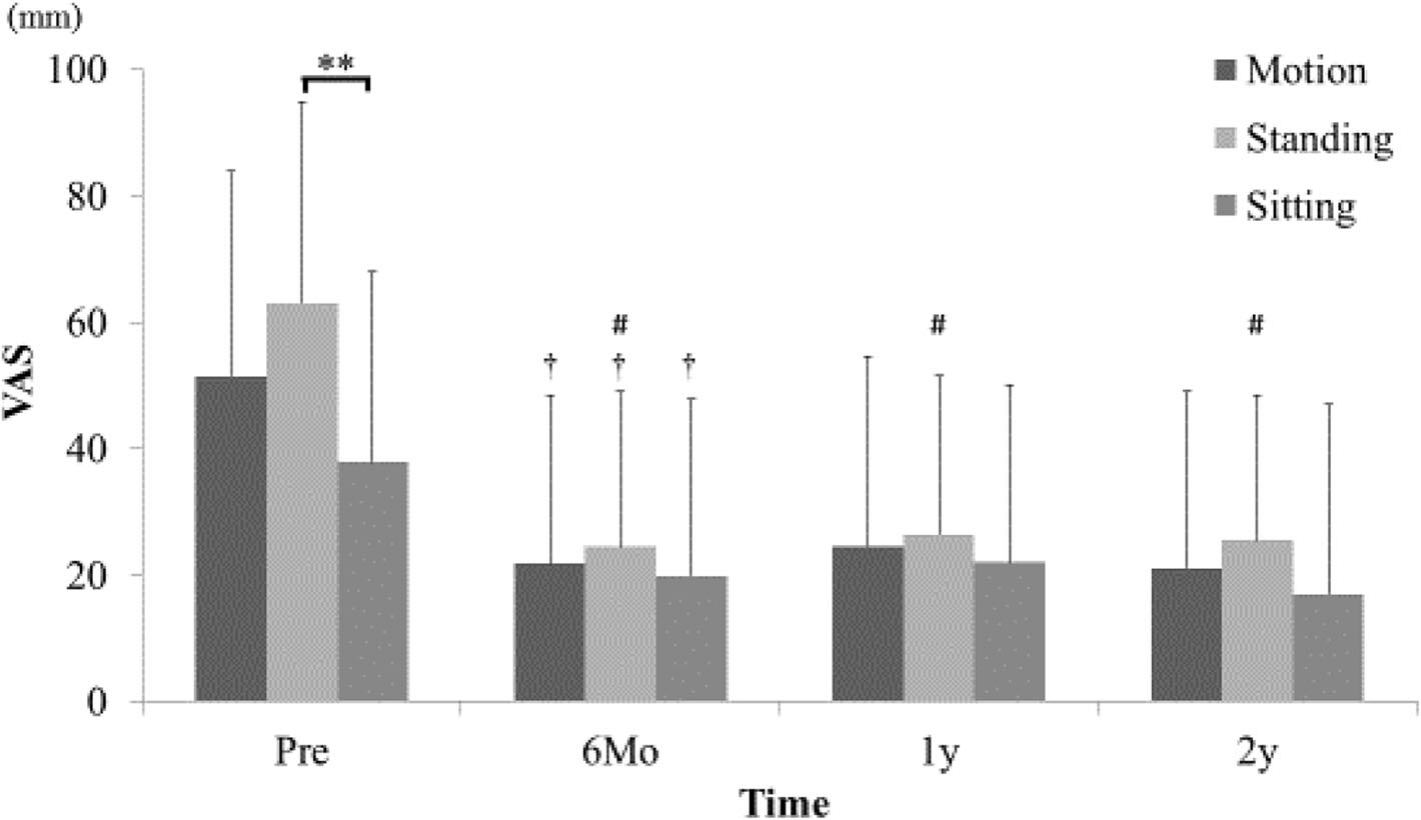
Changes in detailed LBP VAS scores. LBP while standing before surgery was significantly higher than LBP while sitting (**one factor ANOVA, *p* < 0.01). LBP VAS scores in all three postural situations were improved significantly after ULBD surgery (†paired *t* test, *p* < 0.01). Especially, LBP while standing improved significantly after ULBD surgery compared with LBP while sitting (# repeated measure two-factor ANOVA, *p* < 0.05). The level of LBP became almost the same in the three postural situations and this continued until the 2-year follow-up

Detailed LBP VAS before surgery was 51.5 ± 32.4 in motion, 63.0 ± 30.1 while standing, and 37.8 ± 31.8 while sitting; the result showed LBP while standing was significantly higher than LBP while sitting (*p* < 0.01). Compared with that while sitting, the strong LBP while standing was significantly improved by ULBD surgery (*p* < 0.05). The levels of LBP became almost the same for each postural position, and LBP relief was maintained until the 2-year final follow-up (Fig. 3).

**Fig. 4:**
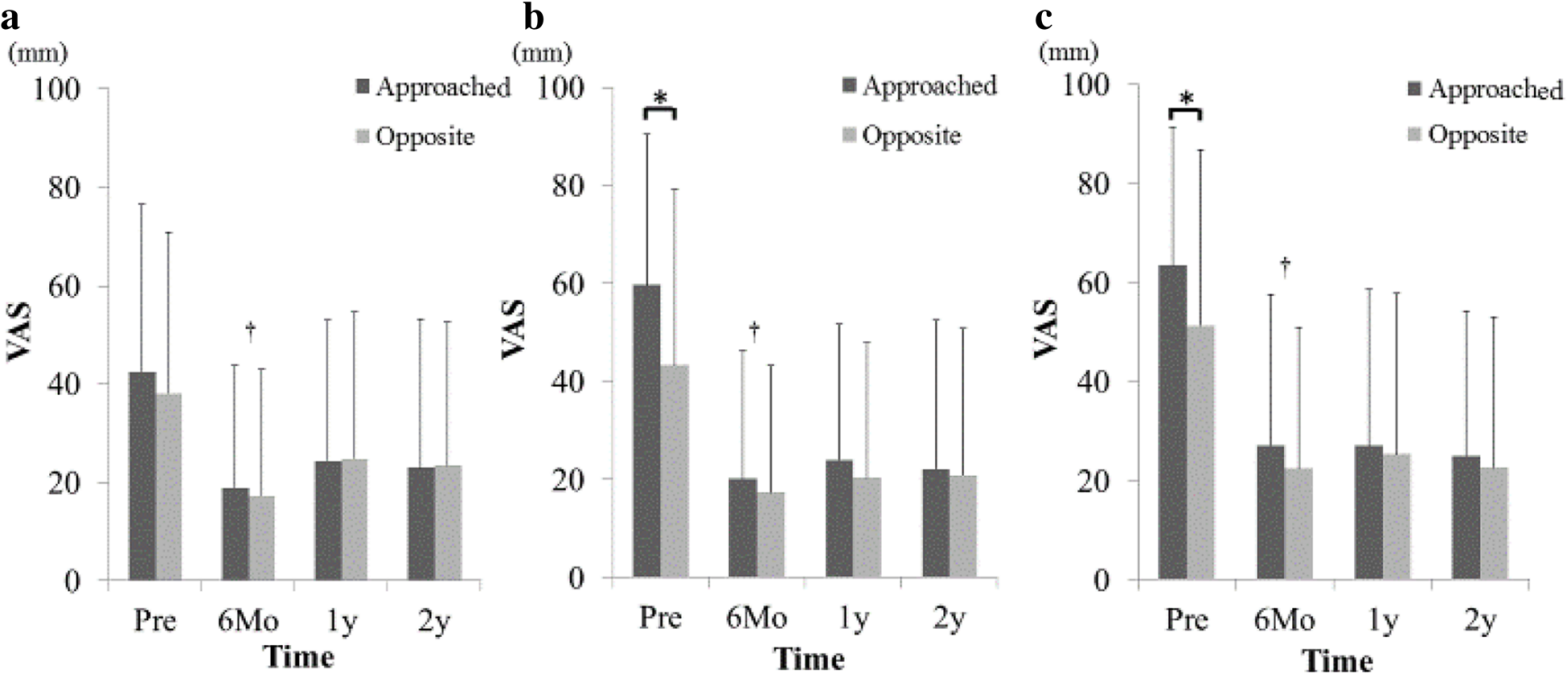
Changes of bilateral VAS scores of LBP (**a**), LEP (**b**), and LEN (**c**). LEP and LEN on the approached side before surgery were significantly greater than that on the opposite side (*p < 0.05). After ULBD surgery, LBP, LEP, and LEN were significantly improved on the approached and opposite sides equally (†paired *t* test, *p* < 0.01)

**Fig. 5:**
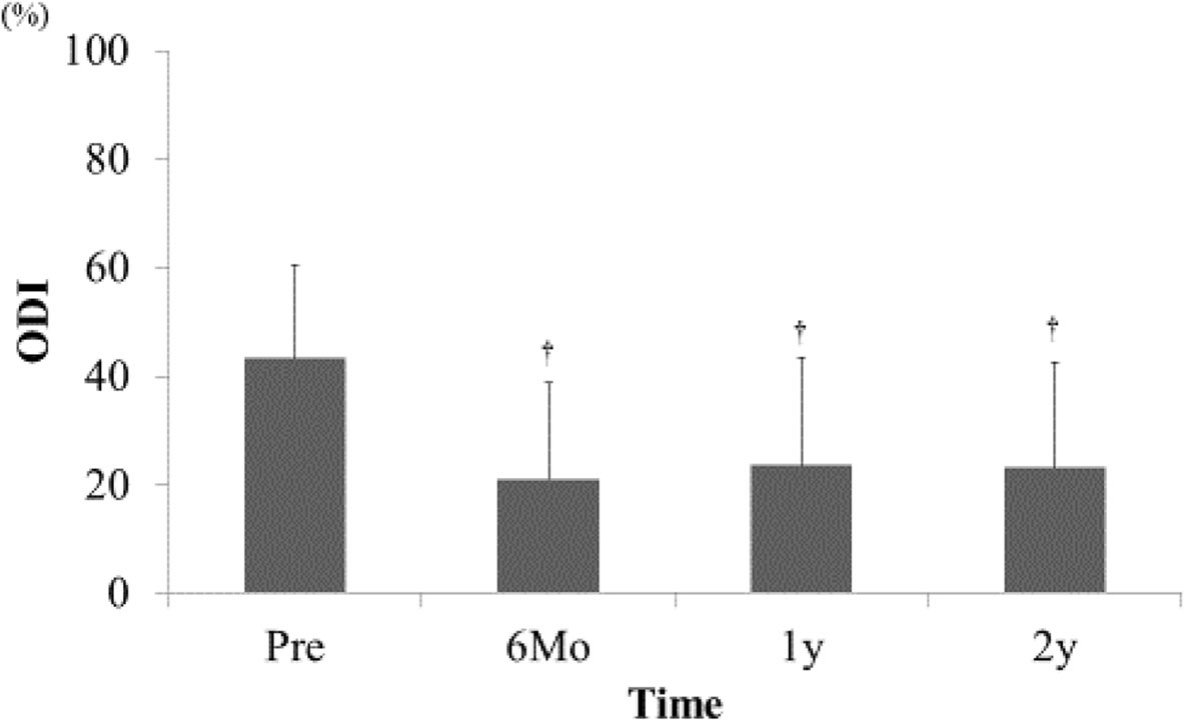
Changes of ODI. ODI was significantly improved by ULBD surgery (†repeated measure single-factor ANOVA, *p* < 0.01)

Bilateral LBP VAS score before surgery was 43.3 ± 38.2 on the approached side and 38.2 ± 31.9 on the opposite side without significant difference. The bilateral LBP was improved significantly on both sides equally, and LBP relief was maintained until the 2-year final follow-up. By contrast, LEP and LEN on the approached side were significantly greater before surgery because the approached side was determined by the patient’s most affected side (LEP approached side: 59.3 ± 30.6, LEP opposite side: 42.3 ± 35.4, *p* < 0.05 / LEN approached side: 63.1 ± 27.7, LEN opposite side: 50.1 ± 35.1, *p* < 0.05). The significant improvements of LEP and LEN were shown to be equal on both sides and like bilateral LBP, were maintained until the 2-year final follow-up (*p* < 0.01, Fig. 4). In the clinical evaluation, ODI showed significant improvement (Fig. 5).

### Evaluation of correlation between X-ray image findings and clinical outcomes

**Fig. 6:**
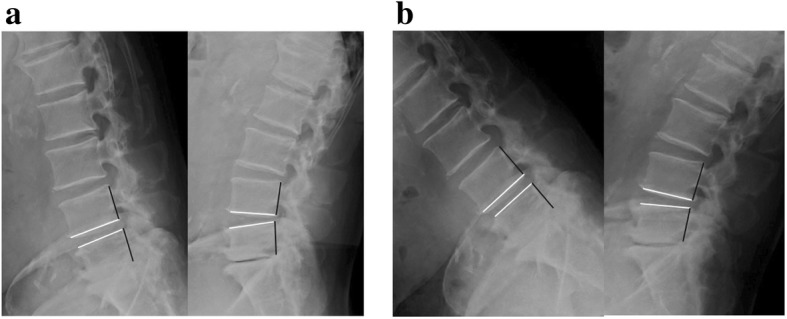
X-ray lateral image at flexion and extension of a representative patient who underwent L4–5 ULBD. **a** Before surgery. **b** Two years after surgery. The white line shows the local ROM and black line shows the translation. The change of local ROM by ULBD is − 2° and the change of translation is 0 in this patient who did not show occurrence of any severe instability after surgery

**Fig. 7:**
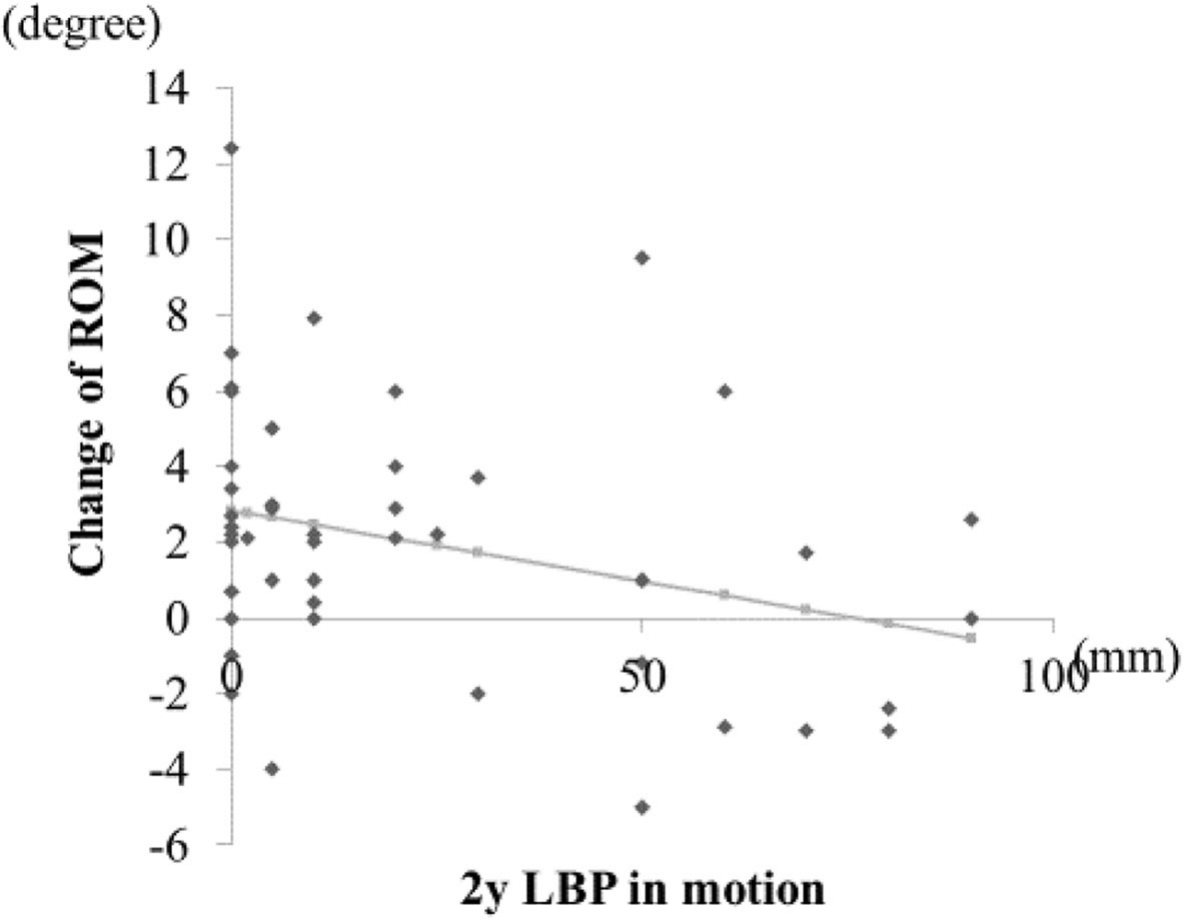
We observed a weak negative correlation between the changes of local ROM and residual LBP in motion at the 2-year final follow-up (*r* = − 0.2993, *y* = 2.838–0.037*x*, *p* = 0.034)

The change of local ROM (°) was 2.05 ± 3.51 (− 5 to 12.4) and the change of local translation (mm) was 0.848 ± 1.87 (− 4.1 to 6.3), showing that severe instability did not occur after surgery (a representative case is shown in Fig. 6). We evaluated the correlation between the residual LBP using bilateral VAS scores in each of the three postures and changes of local ROM. We found a weak negative correlation between residual LBP in motion (2 years after surgery) and changes of local ROM (*r* = − 0.2993, *y* = 2.838–0.037*x*, *p* = 0.034, Fig. 7). There were no significant correlations between residual LBP while standing or sitting (2 years after surgery) and changes of local ROM (data not shown), nor were there any significant correlations between residual LBP in motion, or while standing or sitting, and changes of translation (data not shown).

## Discussion

To our knowledge, this study is the first to evaluate the characteristics and transitions of LBP in patients with LSS treated with decompression surgery. A detailed VAS scoring system for various lumbar disorders was first introduced by Aoki et al. and found to be useful. In an analysis of patients with nonspecific LBP, elderly patients showed significantly lower LBP VAS scores while sitting compared with that in a group of young patients, indicating the possibility of a discogenic factor in younger patients [10]. In early-stage spondylolysis, the LBP VAS score while in motion was significantly higher than that found while standing or sitting [11]. By contrast, although there are some studies that evaluated bilateral LEP VAS scores divided into those on left and right sides [12], there are no studies that measured LBP VAS scores bilaterally.

The noteworthy point of the present study are the findings that LBP in patients with LSS before surgery was significantly greater while standing and the pain was reduced by ULBD surgery, with LBP (and LEP and LEN) improving equally on the approached and opposite side. These findings suggest, at least in part, that radicular LBP is improved by bilateral nerve root decompression surgery, supporting the report by Toyone et al. that LBP is improved by lumbar nerve root decompression surgery [13]. Furthermore, LBP improvement after surgery to decompress the cauda equina may also be found, although signs of cauda equina syndrome do not generally result in LBP. In addition, we speculate the mechanism of the postural and symmetrical LBP improvement as being that the loads to the facet joint and disc bilaterally were improved because of the bilateral LEP relief by the decompression of the bilateral nerve root and cauda equina. Minimally invasive surgery particularly can also result in a favorable outcome for the treatment of patients with chronic LBP and spinal stenosis and our present results also support this finding [14]. By contrast, greater preoperative LBP results in a poorer surgical outcome after the decompression surgery [15]. However, in the decompression surgery just mentioned, a conventional laminectomy was performed and ULBD surgery may result in a more favorable outcome. A study comparing the ULBD with the conventional laminectomy indicated ULBD was superior in VAS of LEP and perioperative opioid use [16]. Another study that compared unilateral with bilateral laminectomy indicated that unilateral laminectomy induces less translational motion because of the preservation of paraspinal muscle and facet joint of opposite side [17]. According to these studies and our present results, ULBD is superior to bilateral conventional laminectomy in its clinical results and X-ray instability due to the preservation of paraspinal muscle and the facet joint on the opposite side. Generally, it is well known that LBP in LSS is multifactorial and that it includes facet pain, discogenic pain, radicular pain, and psychogenic pain [18]. Our detailed three posture LBP VAS suggests that stronger LBP while standing than that while sitting reflects radicular or cauda equina symptoms such as intermediate LBP. By contrast, LBP while in motion may reflect the facet pain, discogenic pain, and radicular pain due to dynamic factors. Ultimately, in this study, LBP in the three postures improved to the same level after ULBD surgery. This may reflect that the intermediate LBP improved by ULBD surgery. In addition, the bilateral VAS scores improved equally on the approached and opposite sides indicating that the loads to facet joint and disc bilaterally were improved because of the bilateral LEP relief by decompression of the bilateral nerve root and cauda equina. For residual LBP, ULBD has less of a clinical outcome benefit than bilateral laminectomy, and this may result from the asymmetrical approach [19]. However, in the present study, ULBD surgery did not worsen the residual LBP, but produced sufficient improvement of LBP on the approached side despite unsymmetrical invasion of the paraspinal muscles and facet joints. Non-neuropathic factors contribute to the LBP of patients with LSS [20]. It is well known that the cause of LBP is multifactorial: deranged discs, facet joints, nerve roots, para-spinal muscles, and psychogenic factors [18]. In the present study, patients with LBP complicated with DLS were excluded to avoid confounders of LBP caused by instability of discs and facet joints. To consider these types of pain, further investigations will be needed.

In the present X-ray image evaluation, severe instability or hypermobility such as severe worsening of local ROM or translation was not found because the spinous ligaments were preserved by ULBD surgery as shown in a representative case in Fig. 6 [5]. Furthermore, a weak negative correlation between residual LBP during motion (2 years after surgery) and changes of local ROM was found. The smaller the residual LBP in motion, the more local ROM was obtained. This finding supports a report that spinal motion was significantly increased in flexion after multilevel fenestration [21]. By contrast, changes of translation were not correlated with residual LBP, especially LBP in motion, which may reflect facet pain unrelated to changes of translation, and may also reflect asymmetrical invasion of paraspinal muscles and facet joints unrelated to the residual LBP.

The present study has some limitations. First, this study is observational and we did not evaluate the bilateral VAS scores of patients who underwent conventional laminectomy. Ideally, a randomized clinical trial that compares conventional laminectomy and ULBD should be conducted to demonstrate causality. However, conventional laminectomy may lead to the residual LBP because of the postoperative instability of decompression segment and ULBD is superior in the clinical outcome [4, 5, 7, 8]. Thus, such study is precluded by ethical constraints. Second, because of its small sample size, the present study includes both single level and multilevel cases. The invasion of the paraspinal muscles and facet joints is different in single level and multilevel cases. However, LBP VAS scores before surgery are unrelated to the surgical procedure and therefore it is feasible that the significantly strong LBP that exists while standing will be improved by ULBD surgery. Study with a larger sample will be needed to improve statistical power. Third, the present study excluded patients with DLS who have unstable vertebra because we wanted to avoid LBP caused by instability of discs and facet joints. If these patients wanted, we performed ULBD surgery for DLS (4 cases in this study). However, we usually recommend transforaminal lumbar interbody fusion for patients with DLS. Further investigation will be needed to evaluate the characteristics and changes of bilateral LBP VAS scores in patients with DLS. Fourth, the present study did not evaluate sagittal alignment. Sagittal imbalance such as pelvic incidence and lumbar lordosis mismatch may be related to postoperative LBP [22]. Using detailed VAS scores, Aoki et al. indicated that sagittal imbalance after the short segment fusion surgery resulted in residual LBP while standing [23]. The main purpose of the present study was to evaluate the characteristics and transitions of LBP using detailed bilateral VAS scores. Thus, we did not evaluate the correlation between residual LBP and sagittal alignment. Investigation of the changes of sagittal alignment will be needed to evaluate further the characteristics of residual LBP.

## Conclusions

ULBD improves LBP while standing, equally on both sides in patients with LCS. The improvement of LBP while standing suggests radicular LBP improved because of decompression surgery. Furthermore, the symmetric improvement of LBP by the ULBD surgery suggests unsymmetrical invasion of the paraspinal muscles and facet joints was unrelated to the residual LBP. When evaluating residual LBP, investigation of sagittal alignment is necessary and further clinical study will be needed to elucidate this point.

## Abbreviations

DLS: degenerative spondylolisthesis, ROM: range of motion
LBP: low back pain
LEN: lower extremity numbness
LEP: lower extremity pain
LSS: lumbar canal stenosis
MRI: magnetic resonance imaging
ODI: Oswestry disability index
ULBD: unilateral laminectomy for bilateral decompression
VAS: Visual analog scale
